# The Hospital World

**Published:** 1910-07-09

**Authors:** 


### The Hospital World.

### Elections and Appointments.

Mr. Cosmo Bonsor has been elected President, and Viscount Goschen Treasurer of Guy's Hospital.

Miss O'Brien, Miss Sibthorpe, Alderman George Healy, J.P., and Mr. Lawrence Mulligan, J.P., have been appointed Visiting Governors for July to the Royal Hospital for Incurables, Aberdeen.

Captain James Foster, Woodcote, has been elected treasurer, and Mr. G. Butler Lloyd was re-elected deputy treasurer of the Salop Infirmary, and Mr. H. J. Allcroft and Colonel Lovatt are to fill the vacancies on the board caused by the deaths of General Herbert and Mr. Spencer Phillips.

Mr. E. T. Dowson and Mr. T. D. Grissell have been re-elected to the Committee of the Beccles Hospital. Captain Barne has been asked to serve in place of the Rev. A. T. G. Thackeray, who has retired from the rectorship of Beccles. His successor when appointed will be elected to the Committee.

The officers of the Malvern Link Dispensary have been re-elected thus: The Rev. A. Day, President; Dr. Dixey, Mr. W. Jones, and Mr. W. Douglas, Vice-Presidents; Mrs. George, Hon. Secretary; Mr. S. L. Wade, Hon. Treasurer. Mr. M. Layton, Mr. H. Littlewood, Mr. W. H. Rowe, and Mr. Gardner have been elected to the Committee, and Mr. A. Littlewood has been reappointed Collector.

Mr. Thomas Cope, J.P., is to be the first President of the Leicester Maternity Hospital, which hitherto has been attached to the Provident Dispensary of the town, but now is to start on a career of its own independently. The other officers of the hospital have been chosen as follows: Sir Edward Wood, Mrs. T. Fielding Johnson, Mrs. G. H. Ellis, and Mr. John E. Faire to be Vice-Presidents. To be Hon. Treasurer, Mr. J. E. Faire; Hon. Consulting Surgeon, Dr. Franklin; Hon. Auditor, Mr. A. H. Hampson; Hon. Financial Secretary, Mr. J. G. Pickard; and Hon. Corresponding and Minute Secretary, Mrs. A. H. Paget.

The following gentlemen and ladies, selected from the donors of £20 taken in rotation, have been appointed vice-presidents of the East Suffolk and Ipswich Hospital: Mr. F. Turner, Mr. W. Bantoft, Mrs. Cranfield, Mr. P. W. Cobbold, Mr. Herbert St. George Cobbold, and Mr. V. D. Colchester. Mr. William Alexander and Mr. A. Gibb have been elected hon. treasurers, and Mr. I. L. Ensor auditor. The board of management is to be as follows: Mr. S. R. Anness, Mr. F. W. Canham, Mr. J. D. Cobbold, Mr. G. Crickmer, Mr. B. W. Elkington, Mr. W. A. Francis, Mr. F. Messent, Mr. A. J. Potter, Mr. Herbert Wright, Mr. Henry P. Henderson, Admiral Pelham Aldrich, and Mr. J. W. Aldous, the last three being new members.

On the proposition of Mr. H. K. Beale, Chairman of the Children's Hospital Committee, the following gentlemen have been appointed a sub-committee to have charge of the money subscribed for the Birmingham Children's Hospital, and the balance of the general fund after paying all charges in connection with the memorial statue (it will be remembered that the statue and hospital schemes are Birmingham's memorial to the late King, for which £10,000 has been subscribed already): The Lord Mayor, Sir John Holder, Bart., Alderman J. H. Lloyd, Mr. J. R. H. Smyth, Mr. W. W. Butler, Mr. Charles Hyde, Mr. J. B. Clarke, Mr. H. F. Harvey, Mr. H. F. Keep, and Mr. A. M. Chance.

### Personalia.

His Majesty the King has become patron of the Norfolk and Norwich Hospital.

The Queen has graciously accorded her patronage to the Chelsea Hospital for Women.

The Rev. R. B. Cooke and Mr. James Haveron, the honorary secretaries of the Royal Victoria Hospital, Belfast, have received the special thanks of the Board of Management for the energy and skill with which they organised the Hospital Saturday Collection. This year the sum received has amounted to some £900.

Sir H. Seymour King, M.P., has been re-elected President and Mr. S. H. Holmes, J.P., Vice-President of the Hull Hospital for Women and Orthopædics. Major C. Judge, J.P., and Mr. A. Jordan have been reappointed Hon. Treasurers, Mr. W. J. Stuart Hon. Secretary, and Mr. A. E. Peasegood Hon. Auditor. Mr. Walsh has been nominated by the Working Men's Committee a member of the Board of Management.

Some time ago it was reported in these columns that the medical superintendent of the Melbourne General Hospital had reduced the cost of surgical dressings, medical gauze, etc., by the employment of women, instead of men, as dressers. The enormous reduction of £963 has been effected during the first quarter of this year by the appointment of a house matron and putting women cooks in the kitchen. Dr. Mackay brought down dressings from £434 to £200, the house matron clipped £125 off the butter bill, £113 off eggs, £76 off milk, £64 off groceries, £55 off vegetables. The House Committee have given every assistance, and now propose to do away with the system of calling for tenders for food supplies. All this was done during a term when the hospital was busier than ever before, and this without suffering or deprivation to either patients or staff. As the friends of patients are always anxious to bring in delicacies, generally of the most undesirable kind, the suggestion is made to them now that eggs would be acceptable, so a great many are brought. But the patient whose friends do not bring him eggs still gets these when he requires them.

At the last meeting of the House Committee of the Leicester Infirmary opportunity was taken to present Miss Sherlock (Sister Lena) with a crocodile leather dressing-case and silver fittings, in appreciation of the valuable services she has given to the infirmary during the long period of twenty-five years. Mr. T. Fielding Johnson, jun., presided, and asked Dr. Pratt, who had kindly undertaken the hon. secretaryship of the presentation committee, to present the case. It bore a silver plate on the cover with a suitable inscription. But this was not all, for the members of the Leicester Infirmary Nurses' League (of which Miss Sherlock for several years had been the hon. secretary) have presented Sister Lena with a gold keyless watch and a purse of money as a mark of their affection. This presentation was made by Miss G. A. Rogers, President of the League. It has been a long and intimate connection, for Miss Sherlock was trained at the Leicester Infirmary, and in its wards her long service has been wholly spent. She leaves to become the matron of the Home of Recovery at Hunstanton, in connection with Addenbrooke's Hospital, and begins her duties in the middle of July with the best wishes of all her friends at Leicester.

